# Fingerprint Analysis and Quantitative Determination of Fourteen Active Components in the Traditional Chinese Medicinal Preparation *Feiyang*changweiyan Capsule by HPLC-DAD-ESI-MS/MS

**DOI:** 10.22037/ijpr.2019.1100649

**Published:** 2019

**Authors:** Dan Zhou, Qian Yang, Zhe Yu, Ying Chang, Tian Tian, Zhi-Hui Shi, Hong-Liang Dong, Hua Li, Jun Guo, Si-Wang Wang

**Affiliations:** a *Department of Natural Medicine, School of Pharmacy, Fourth Military Medical University, Xi’an, China.*; b *Department of Pharmacy, Ninth Hospital of Xi’an, Xi’an, China.*; c *Department of Pharmaceutical Analysis, School of Pharmacy, Fourth Military Medical University, Xi’an, China. *; d *Department of Pharmacy, Northwest Women and Children Hospital, Xi’an, China.*; e *Jun Bi Sha Pharmaceutical Co. Ltd. Xianyang, China.*; f *Department of Radiology, School of Stomatology, Fourth Military Medical University, Xi’an, China*.; g *College of Life Science and Medicine, Northwest University, Xi'an, China*.; 1 D. Z., Q. Y., Z. Y. and Y. C. contributed equally to thiswork

**Keywords:** HPLC-DAD-ESI-MS/MS, Quantitative analysis, Traditional Chinese medicine, *FeiYang*changweiyan capsule, Fingerprint

## Abstract

*FeiYang*changweiyan capsule (FY capsule), a traditional Chinese medicinal preparation consisting of three medicinal herbs, has been used to treat bacterial dysentery, acute, and chronic gastroenteritis for several decades. In this study, a novel, convenient, accurate, and valid method was developed by using high-performance liquid chromatography (HPLC) coupled with diode array detection (DAD) to obtain a chromatographic fingerprint of *FeiYang*changweiyan capsule (FY capsule). Then, fourteen peaks were identified according to MS/MS fragmentation behavior of the reference standards by using HPLC-DAD-ESI-MS/MS analysis. At the same time, the fingerprint similarity was calculated and the contents of known ingredients were also determined simultaneously. The result demonstrated that the HPLC fingerprint combining similarity evaluation and quantification analysis can be successfully applied to control the quality of FY capsule.

## Introduction

Traditional Chinese medicines (TCMs) are playing an important role in Chinese healthcare system and will have prospect future all over the world due to their reliable therapeutic efficacy (1, 2). However, the complexity and ambiguity of compositions have restricted the development of TCM, which has become the bottleneck that obstructs the broad (3). Therefore, quality control is vital to judge the merits of the authenticity (4).

Fingerprint which shows chemical information is wildly used for quality control of traditional Chinese medicine (TCM) (5). And World Health Organization (WHO), U.S. Food and Drug Administration (FDA) and State Food and Drug Administration of China (SFDA) suggest that fingerprint technology can be used in the process of establishing quality standards for TCM (6). The chromatographic methods including HPLC, GC, HPTLC, and CE are recognized as the routine analysis methods for the identification and qualification of herbal medicines (7-9). Currently, selection of a single or a few specific components as markers for quality assessments is a widely applied strategy (10). However, according to TCM theory, all the medicinal compositions play roles in therapeutic effects. Unlike synthetic drugs, the therapeutic effects of TCM and their preparations exert curative effects based on the synergic effects of their multi-components and multi-targets (11, 12). Thus, a more comprehensive and global view, which could cover most of the active chemical constituents, is valuable for the quality control of traditional medicine (13).

*FeiYang*changweiyan capsule (FY capsule), composed of three traditional Chinese medicines *Euphorbia hirta L. *(Fei Yang Cao), *Polygunum chinense L. *(Huo Tan Mu),* Ilex rotunda Thunb. *(Jiu Bi Ying), has been officially recorded in Drug Standard of Ministry of Public Health of the People’s Republic of China for the treatment of bacillary dysentery and gastroenteritis. Despite the largely clinical use of FY capsule, quality control studies for the constituents of the formula are limited. Currently, there are a few analytical methods available for evaluating the quality of FY capsule, which are only able to determine one or two active ingredients (14). According to the literature (15-20), the major constituents in FY capsule, such as gallic acid (GA), quercitrin (QI), quercetin (QE), caffeic acid (CA), and isoquercitrin (IQ) from *Euphorbia hirta L.*, protocatechuic acid (PA), caffeic acid (CA), myricetin (MY) and rosmarinic acid (RA) from *Polygunum chinense L.* and lithospermic acid (LA), syringic acid (SA), methyl gallate (MG), ellagic acid (EA), Syringin (SY) and chlorogenic acid (CHA) from *Ilex rotunda Thunb.*, were reported significant pharmacological activities, especially anti-inflammatory, anticancer, anti-influenza virus, antiplatelet aggregation, antimicrobial, and anti-diarrheal (21-26). Although HPLC methods have been applied to determine some of the constituents in crude drugs and Chinese patented medicines, no chromatographic fingerprints for the quality control of the compound prescription as well as no analytical method has been reported for simultaneous determination of the fourteen major constituents in FY capsule until now (27-29). This is extremely adverse to constituent illustration and active component screening. Thus, with a view to the further development of FY capsule, it is necessary to clarify its composition and establish quality standards. 

In the present study, chromatography fingerprint of FY capsule was established and an HPLC-DAD-ESI-MS/MS method was developed for the identification and simultaneous determination of the fourteen components in FY capsule. To the best of our knowledge, it is the first time that the main constituents in FY capsule have been identified and simultaneously determined. From these results, the proposed method in this paper is particularly suitable for the routine analysis of FY capsule and its quality control.

## Experimental

*Chemicals and reagents*

Methanol (HPLC grade) was purchased from Honeywell (Muskegon, MI, USA). Glacial acetic acid (HPLC grade) was purchased from Mallinckrodt Baker (Phillipsburg, NJ, USA). Standards including GA, QI, QE, IQ, PA, CA, MY, RA, LA, SA, MG, EA, SY, and CHA were purchased from National Institute for the Control of Pharmaceutical and Biological Products (Beijing, China). The purity of these compounds was determined to be more than 98% by normalization of the peak areas detected by HPLC, and was showed very stable in methanol solution. The deionized water was prepared from Millipore water purification system (Milford, MA, USA) and filtered with a 0.22 μm membrane. Other reagents were all of analytical grade. FY capsules were provided by Shaanxi Jun Bi Sha pharmaceutical co., LTD.

**Figure 1:**
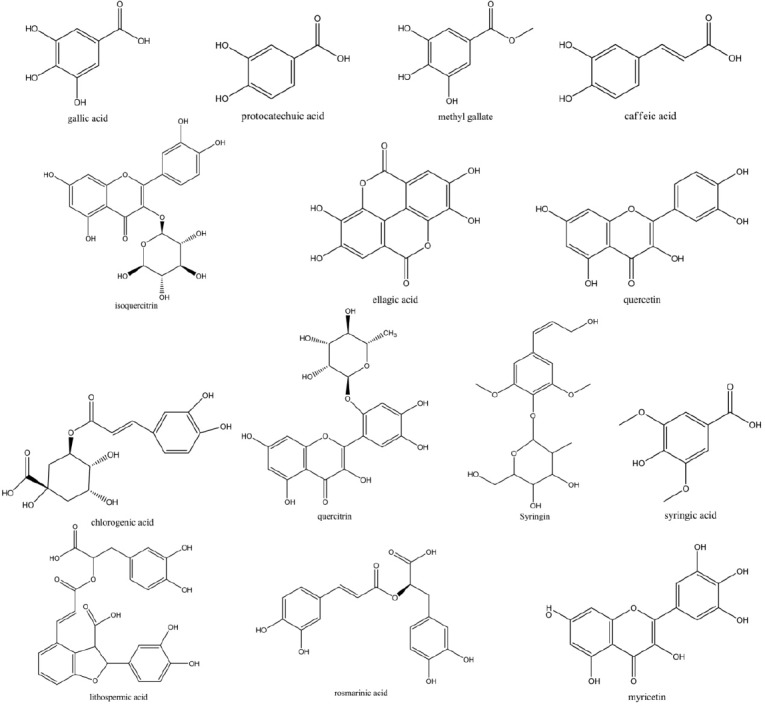
Chemical structures of the 14 active components contained in FY capsule

**Figure 2:**
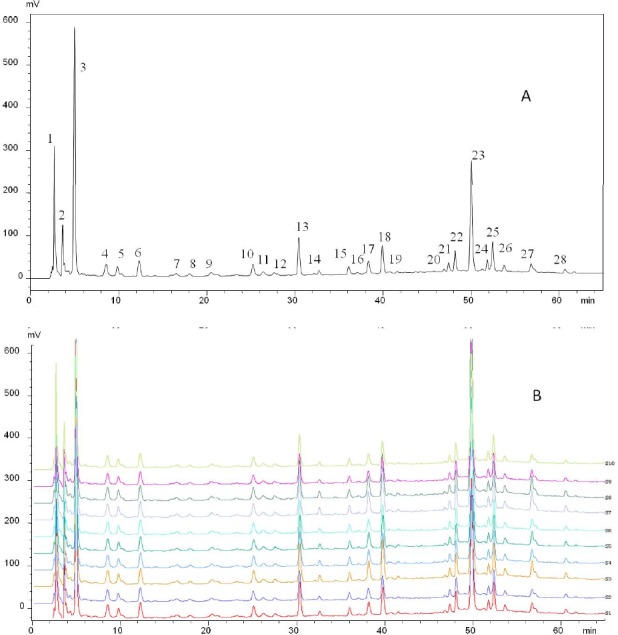
Fingerprint of FY capsule. (A) The representative standard fingerprint. (B) The similarity of the fingerprint of 10 samples derived with CASE software

**Figure 3:**
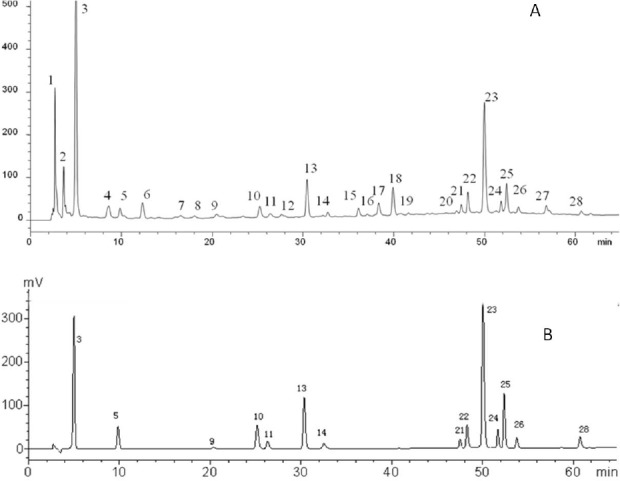
Typical chromatograms of (A) FY capsule and (B) the standard mixture at 280 nm. (3) Gallic acid; (5) protocatechuic acid; (9) Methyl gallate; (10) Chlorogenic acid; (11) Caffeic acid; (13) Syringin; (14) Syringic acid; (21) Myricetin; (22) Isoquercitrin; (23) Ellagic acid; (24) Lithospermic acid; (25) Rosmarinic acid; (26) Quercitrin; (28) Quercetin

**Table 1:** Analytical method validation results for the fingerprint analysis

**Peak no.**	**RSD of relative retention time (%)**			**RSD of relative peak area (%)**		
	**Precision (n = 5)**	**Reproducibility (n = 5)**	**Stability (n = 7)**	**Precision (n = 5)**	**Reproducibility (n = 5)**	**Stability (n = 7)**
1	0.15	0.21	0.11	3.12	2.18	3.64
2	0.21	0.14	0.14	2.51	2.64	1.34
3	0.28	0.11	0.16	2.79	2.12	2.66
4	0.36	0.25	0.32	2.19	1.38	3.29
5	0.42	0.15	0.19	4.60	1.61	1.72
6	0.28	0.31	0.06	2.31	3.51	2.71
7	0.31	0.28	0.17	3.25	4.62	4.31
8	0.15	0.27	0.08	2.69	2.37	2.67
9	0.35	0.15	0.17	3.11	2.85	3.33
10	0.37	0.10	0.11	3.24	4.86	1.08
11	0.26	0.06	0.10	2.47	1.51	3.00
12	0.16	0.26	0.14	3.11	2.64	1.67
13	0.09	0.05	0.07	2.38	1.47	1.75
14	0.17	0.04	0.06	3.12	2.99	1.66
15	0.21	0.08	0.53	1.21	2.65	3.61
16	0.19	0.16	0.23	3.21	1.26	3.67
17	0.37	0.05	0.21	1.69	4.21	4.12
18	0.18	0.26	0.06	4.11	3.67	3.91
19	0.25	0.05	0.28	2.37	2.35	1.26
20	0.09	0.24	0.21	1.54	3.67	2.67
21	0.03	0.03	0.01	4.41	4.54	2.08
22	0.03	0.03	0.91	4.31	3.72	2.82
23	0.08	0.05	0.03	2.73	1.83	3.74
24	0.03	0.58	0.01	4.11	4.42	3.89
25	0.02	0.71	0.01	4.70	3.28	2.55
26	0.02	0.02	0.01	2.17	4.95	4.43
27	0.16	0.31	0.16	2.34	1.38	3.61
28	0.00	0.00	0.00	0.00	0.00	0.00

**Table 2:** Similarities of chromatograms of 10 batches

**Similarity**	**S1**	**S2**	**S3**	**S4**	**S5**	**S6**	**S7**	**S8**	**S9**	**S10**	**R**
S1	1										
S2	0.996	1									
S3	0.996	0.994	1								
S4	0.993	0.995	0.998	1							
S5	0.995	0.994	1	0.998	1						
S6	0.998	0.994	0.991	0.997	0.991	1					
S7	0.996	0.995	0.995	0.999	0.995	0.995	1				
S8	0.998	0.991	0.995	0.999	0.995	0.995	0.999	1			
S9	0.992	0.995	0.996	0.999	0.997	0.997	0.997	0.996	1		
S10	0.999	0.998	0.998	0.998	0.997	0.995	0.994	0.996	0.997	1	
R	0.985	0.994	0.999	1	0.999	0.996	0.999	0.999	0.999	0.997	1

**Table 3:** Characterization of ingredients of FY capsule by HPLC–MS/MS(3,5,9,10,11,13,14,21,22,23,24,25,26,28 was the peak with [M-H]-)

**Peak No.**	**t** **R ** **(min)**	**Formula**	**Measured mass (m/z)**	**Calculated mass (m/z)**	**Δ** **(ppm)**	**Compound**
3	4.98	C7H6O5	169.0131	169.0142	-6.5	gallic acid
5	9.78	C7H6O4	153.0191	153.0193	-1.3	protocatechuic acid
9	20.26	C8H8O5	183.0297	183.0299	-1.09	methyl gallate
10	25.05	C16H18O9	353.0883	353.0878	1.42	chlorogenic acid
11	26.17	C9H8O4	179.0342	179.035	-4.47	caffeic acid
13	30.26	C17H24O9	371.1361	371.1348	3.5	syringin
14	32.04	C9H10O5	197.0448	197.0455	-3.55	syringic acid
21	46.81	C21H20O12	463.0887	463.0882	1.08	myricetin
22	48.72	C21H20O12	463.0862	463.0882	-4.32	isoquercitrin
23	49.92	C14H6O8	301.001	300.999	6.65	ellagic acid
24	52.22	C27H22O12	537.1002	537.1038	-6.7	lithospermic acid
25	52.90	C18H16O8	359.0756	359.0772	-4.56	rosmarinic acid
26	53.68	C21H20O11	447.0907	447.0933	-5.82	quercitrin
28	60.61	C15H10O7	301.0354	301.0354	0	quercetin

**Table 4:** Regression equation, linear range and LODs of the developed method

**Constituent**	**Regression equation** a	**Correlation coefficient (r** **2** **)**	**Linearity range (μgmL** **-1** **)**	**LOD (μgmL** **-1** **)**
gallic acid	y = 31293x + 62560	R2 = 0.9999	3.90-500.00	0.06
protocatechuic acid	y = 69948x - 4915.5	R2 = 0.9997	0.39-50.00	0.05
methyl gallate	y = 31368x - 1179.8	R2 = 0.9998	0.78-10.00	0.09
chlorogenic acid	y = 28169x + 8765.6	R2 = 0.9999	1.17-150.00	0.07
caffeic acid	y = 43126x + 4696.3	R2 = 0.9995	0.23-30.00	0.05
syringin	y = 38657x + 8051	R2 = 1.0000	1.56-200.00	0.06
syringic acid	y = 25756x - 1299.5	R2 = 0.9999	0.23-30.00	0.03
myricetin	y = 42629x - 7886.6	R2 = 0.9998	0.23-30.00	0.07
isoquercitrin	y = 53952x - 6171.6	R2 = 0.9991	0.47-60.00	0.06
ellagic acid	y = 193209x + 159781	R2 = 0.9997	0.78-100.00	0.04
lithospermic acid	y = 32432x - 447.8	R2 = 1.0000	0.51-65.00	0.07
rosmarinic acid	y = 25856x + 9391.3	R2 = 1.0000	1.95-250.00	0.06
quercitrin	y = 51800x - 820.42	R2 = 0.9995	0.20-25.00	0.02
quercetin	y = 68657x - 7541.2	R2 = 0.9998	0.20-25.00	0.02

a y: peak area of components; x: concentration of components.

**Table 5:** The precision and recovery datas of the proposed HPLC method

**Components**	**Nominal** **Concentration (μgmL** ^-1^ **)**	**Precision**				**Recovery**
		**Intra-day (n = 5)**		**Inter-day (n = 5)**		
		**Mean ± SD (μgmL** ^-1^ **)**	**RSD (%)**	**Mean ± SD (μgmL** ^-1^ **)**	**RSD(%)**	**Mean ± SD (%)**
gallic acid	250	251.00 ± 1.59	0.58	251.90 ± 1.09	0.74	102.21 ± 1.01
	31.25	31.67 ± 0.08	0.24	31.51 ± 0.14	0.04	99.21 ± 0.54
	7.81	7.71 ± 0.02	0.31	7.81 ± 0.12	0.25	99.61 ± 0.23
protocatechuic acid	25	25.42 ± 0.11	0.40	25.31 ± 0.16	0.61	100.25 ± 2.14
	3.13	3.17 ± 0.04	0.28	3.32 ± 0.04	0.12	101.34 ± 0.91
	0.78	0.78 ± 0.03	0.31	0.78 ± 0.05	0.26	99.31 ± 1.06
methyl gallate	5	5.04 ± 0.24	0.48	5.17 ± 0.14	0.28	101.37 ± 1.12
	0.63	0.65 ± 0.06	0.81	0.64 ± 0.06	0.12	99.32 ± 1.14
	0.16	0.15 ± 0.04	0.21	0.17 ± 0.06	0.27	101.37 ± 0.92
chlorogenic acid	75	75.01 ± 0.18	0.23	75.42 ± 0.78	0.15	99.37 ± 1.10
	9.38	9.52 ± 0.07	0.11	9.48 ± 0.11	0.12	99.83 ± 1.24
	2.34	2.14 ± 0.04	0.19	2.24 ± 0.14	0.17	99.98 ± 0.91
caffeic acid	15	15.84 ± 0.19	0.21	15.44 ± 0.32	0.02	99.34 ± 1.24
	1.88	1.82 ± 0.04	0.19	1.90 ± 0.29	0.14	99.71 ± 0.66
	0.47	0.45 ± 0.02	0.57	0.46 ± 0.25	0.41	100.37 ± 0.97
syringin	100	101.76 ± 0.46	0.44	100.89 ± 0.69	0.66	100.27 ± 1.14
	12.5	12.53 ± 0.05	0.37	12.85 ± 0.09	0.07	101.23 ± 1.17
	3.13	3.06 ± 0.03	0.01	3.05 ± 0.02	0.09	99.31 ± 0.94
syringic acid	15	15.32 ± 0.44	0.86	15.60 ± 0.32	0.21	101.24 ± 1.09
	1.88	1.87 ± 0.03	0.13	2.00 ± 0.02	0.14	99.64 ± 1.42
	0.47	0.48 ± 0.02	0.36	0.46 ± 0.14	0.21	99.87 ± 0.72
myricetin	15	15.53 ± 0.08	0.49	15.58 ± 0.21	0.13	99.34 ± 1.25
	1.88	1.89 ± 0.02	0.24	2.04 ± 0.04	0.21	101.32 ± 1.09
	0.47	0.47 ± 0.03	0.36	0.47 ± 0.14	0.24	99.31 ± 0.97
isoquercitrin	30	30.16 ± 0.09	0.31	30.57 ± 0.84	0.27	101.28 ± 0.64
	3.75	3.73 ± 0.08	0.22	3.79 ± 0.14	0.37	97.81 ± 0.61
	0.94	1.05 ± 0.02	0.2	1.04 ± 0.04	0.24	98.69 ± 0.93
ellagic acid	50	50.12 ± 0.58	0.04	50.83 ± 0.59	0.16	99.35 ± 1.05
	6.25	6.15 ± 0.05	0.75	6.31 ± 0.38	0.64	101.32 ± 1.27
	1.56	1.50 ± 0.02	0.14	1.57 ± 0.03	0.15	100.25 ± 0.95
lithospermic acid	32.5	32.25 ± 0.08	0.22	32.34 ± 0.28	0.24	102.28 ± 1.31
	4.06	4.17 ± 0.03	0.63	4.13 ± 0.06	0.15	99.67 ± 1.62
	1.02	1.07 ± 0.02	0.17	1.07 ± 0.01	0.09	99.37 ± 0.98
rosmarinic acid	125	126.11 ± 0.18	0.14	125.19 ± 0.96	0.17	100.93 ± 0.71
	15.63	15.95 ± 0.04	0.25	15.85 ± 0.10	0.06	101.85 ± 1.35
	3.91	3.96 ± 0.03	0.13	3.85 ± 0.10	0.28	99.61 ± 0.68
quercitrin	12.5	12.13 ± 0.04	0.29	12.63 ± 0.54	0.16	99.76 ± 0.94
	1.56	1.62 ± 0.01	0.13	1.62 ± 0.01	0.09	100.92 ± 1.38
	0.39	0.41 ± 0.01	0.21	0.40 ± 0.02	0.70	99.84 ± 0.75
quercetin	12.5	12.63 ± 0.32	0.25	12.53 ± 0.44	0.14	100.47 ± 1.40
	1.56	1.58 ± 0.07	0.46	1.53 ± 0.06	0.41	99.35 ± 0.64
	0.39	0.41 ± 0.02	0.23	0.40 ± 0.01	0.38	102.54 ± 0.91

**Table 6:** Content of the fourteen active components in ten batches of FY capsule

**Components**	**Content (n = 3, mean ± SD, μg** ^.^ **mL** ^-1^ **)**									
	**S1**	**S2**	**S3**	**S4**	**S5**	**S6**	**S7**	**S8**	**S9**	**S10**
gallic acid	252.17 ± 6.35	263.12 ± 8.34	248.21 ± 12.63	267.23 ± 15.09	270.08 ± 13.35	254.71 ± 12.13	246.53 ± 14.31	264.31 ± 12.31	272.64 ± 13.28	257.19 ± 12.57
protocatechuic acid	4.85 ± 0.23	5.31 ± 0.66	5.06 ± 0.39	5.19 ± 0.39	5.29 ± 0.23	4.62 ± 0.59	4.51 ± 0.34	5.21 ± 0.62	4.95 ± 0.41	4.81 ± 0.36
methyl gallate	4.05 ± 0.23	4.09 ± 0.68	4.00 ± 0.73	4.70 ± 0.65	4.35 ± 0.44	4.23 ± 0.40	4.08 ± 0.27	4.51 ± 0.34	4.98 ± 0.25	5.01 ± 0.29
chlorogenic acid	17.98 ± 2.01	18.92 ± 2.60	17.87 ± 1.56	19.52 ± 2.21	20.29 ± 2.30	16.83 ± 2.26	21.32 ± 2.10	18.35 ± 1.98	20.16 ± 1.35	19.25 ± 1.24
caffeic acid	4.46 ± 0.35	4.67 ± 0.87	4.46+0.24	4.89 ± 0.33	4.90 ± 0.34	4.29 ± 0.47	4.95 ± 0.75	5.01 ± 0.56	4.45 ± 0.41	4.46 ± 0.38
syringin	37.40 ± 3.52	39.51 ± 6.26	37.67 ± 3.53	41.11 ± 4.28	40.87 ± 3.78	38.48 ± 4.13	41.23 ± 4.21	40.15 ± 4.34	37.29 ± 3.21	40.15 ± 3.61
syringic acid	6.89 ± 0.21	6.85 ± 1.80	6.89 ± 1.46	7.92 ± 1.50	8.28 ± 1.82	6.88 ± 1.65	7.77 ± 1.20	7.65 ± 1.24	7.35 ± 1.24	6.98 ± 1.64
myricetin	6.51 ± 0.49	6.28 ± 1.28	6.61 ± 0.48	7.02 ± 0.84	6.97 ± 0.56	5.78 ± 0.79	6.61 ± 0.54	6.74 ± 0.57	6.51 ± 0.67	6.74 ± 0.36
isoquercitrin	12.02 ± 1.24	12.92 ± 1.37	12.36 ± 0.91	13.31 ± 1.31	13.29 ± 1.28	11.41 ± 2.21	12.34 ± 1.26	13.21 ± 1.62	12.98 ± 1.74	13.24 ± 1.52
ellagic acid	28.03 ± 2.53	31.48 ± 3.26	29.78 ± 4.58	32.41 ± 3.86	32.62 ± 4.04	28.32 ± 3.24	30.21 ± 2.21	31.29 ± 2.14	29.34 ± 2.13	30.15 ± 2.81
lithospermic acid	8.92 ± 0.63	9.18 ± 0.91	8.70 ± 0.39	9.44 ± 0.58	9.44 ± 0.57	8.31 ± 0.65	9.02 ± 1.02	9.31 ± 0.95	9.21 ± 0.61	8.98 ± 0.67
rosmarinic acid	37.42 ± 2.68	39.99 ± 3.93	37.67 ± 2.13	40.75 ± 0.92	41.35 ± 3.86	37.35 ± 1.24	40.15 ± 1.29	38.91 ± 2.19	39.61 ± 1.24	41.31 ± 2.98
quercitrin	4.50 ± 0.55	4.84 ± 1.21	4.65 ± 0.63	5.24 ± 0.79	5.21 ± 0.68	5.01 ± 0.64	5.35 ± 0.21	4.95 ± 0.34	4.87 ± 0.42	5.21 ± 0.57
quercetin	2.77 ± 0.46	3.05 ± 0.95	3.05 ± 0.47	3.50 ± 0.76	3.39 ± 0.56	2.98 ± 0.46	3.14 ± 0.34	3.45 ± 0.24	3.14 ± 0.21	3.34 ± 0.31

*HPLC instrumentation and chromatographic condition*

All analyses were performed on an Agilent 1200 HPLC system (Agilent Technologies, USA), equipped with a quaternary pump, an online degasser, and a column temperature controller, coupled with an DAD (Alltech Associates, USA) as the detector. All separations were carried on an Agilent Zorbax SB-C_18_ reserved-phase column (250 mm × 4.6 mm, 5 μm, Agilent Corporation). The mobile phase included (A): methanol and (B): 2% glacial acetic acid aqueous solution with gradient elution (0–10 min, 5% A; 10–30 min, 5–20% A; 30–50 min, 20–40% A; 50–65 min, 40–50% A;). There was a 10 min re-equilibration duration between individual runs. The flow rate of the mobile phase was 1.0 mL·min^−1^ and the injection volume was 20 μL. The column temperature was maintained at 40 °C. The components were quantified based on peak areas at the maximum wavelength in their UV spectrum.

*HPLC-ESI-MS/MS instrumentation and chromatographic condition*

The HPLC-ESI-MS/MS system was equipped with Agilent 6520 Q-TOF mass spectrometer and Agilent 1200 HPLC system (Agilent Technologies, USA). Efficient and symmetrical peaks were obtained at a flow rate of 0.8 mL·min^−1^ with a sample injection volume of 10 μL. 

The mobile phase and gradient elution were the same as that described in Section 2.2.1. The mass spectrometry detector (MSD) was operated in the negative ion mode with an ESI source (ESI-). The interface and MSD parameters were as follows: nebulizer pressure, 35 psi (N_2_); dry gas, N_2_ (8 L·min^−1^); dry gas temperature, 350 °C; spray capillary voltage, 4000 V; skimmer voltage, 65 V; fragmentor voltage, 190 V; octopole RF, 750 V. mass range, m/z 100–1100. The Mass Hunter Data Acquisition Workstation Software was applied to system operation and data collection. A reference solution was delivered using an external quaternary pump. This solution contains the following internal mass calibrants: purine (C_5_H_4_N_4_) at m/z 121.050873 and HP-921 [hexakis-(1 *H*,1*H*,3 *H* -tetrafluoro-pentoxy) phosphazene] (C_18_H_18_O_6_N_3_P_3_F_24_) at m/z 922.009798. The instrument provides a mass resolving power of 30,000 ± 500 (m/z 1522). 

The stability of mass accuracy was checked daily, and if values went above 2 ppm error, then the instrument was re-calibrated. When the instrument was operated in MS-MS mode, the isolation width was set at medium (m/z~4) and collision energies of 10, 25, and 40 eV were used.

*Preparation of standard solutions*

A standard stock solution containing the fourteen components (GA, QI, QE, IQ, PA, CA, MY, RA, LA, SA, MG, EA, SY, CHA) was prepared in 50% methanol aqueous solution. Working standard solutions, containing the fourteen compounds were prepared by appropriate dilution of the stock solution. All stock and working standard solutions were stored in brown bottles at 4 °C before analysis. 

*Preparation of sample solutions*

The content of FY capsule (0.5 g) was weighed precisely and dissolved in 50 mL 50% methanol aqueous solution. Then the solution was extracted with ultrasonic for 30 min, settled to the volume of 50 mL. The solution was centrifuged at 12,000×g for 15 min and was filtered with a 0.22 μm microporous membrane prior to analysis. Aliquot (20 μL) of sample solution was injected into the HPLC system for analysis.

## Results and Discussion

*Optimization of HPLC conditions*

Optimization of the separation conditions for HPLC analysis was performed including a suitable chromatographic column, the mobile phase composition, gradient elution program, and detection wavelength. To achieve the good separation within shorter time, several trials were tried which included three kinds of C_18_ reversed-phase columns (Agilent Zorbax SB-C_18_, BDS-Hypersil C_18_, Luna C_18_) and different gradient elution systems of methanol–water and aceltonitrie–water with different modifiers including phosphoric acid, glacial acetic acid, formic acid, and formic acid solutions adjusted by ammonia or triethylamine with different pH values. The results indicated that Agilent Zorbax SB-C_18_ column with the methanol–2% glacial acetic acid solution system using gradient elution was selected as the preferred chromatographic conditions. The flow rate was 0.8 mL/min and the column temperature was maintained at 40 °C. The DAD was employed at the wavelength range from 190 nm to 400 nm for obtaining a sufficient number of detectable peaks. The structures of fourteen components were shown in Figure 1. On the ultraviolet spectra with chromatograms of HPLC-UV of fourteen target compounds in FY capsule, maximum absorbance values around 254 nm and 280 nm were observed. More detectable peaks could be obtained and the baseline was well improved around 254 nm. Hence, characteristic chromatographic patterns were obtained by using 254 nm as the detection wavelength. Optimal HPLC condition used in this study was shown in Section 2.2.1.

*Method validation of the fingerprints*

To obtain a stable and repeatable chromatographic fingerprint of FY capsule for quality control, the precision, reproducibility, and stability of the method used were expressed by the RSD value of the average relative retention times (RRT) and relative peak areas (RPA) of the 28 common characteristic peaks with respect to the reference peak (peak 28). The method precision was obtained by successive analysis of the same sample solution five times, and the results demonstrated that the RSDs of precision were not exceeding 0.42% for the RRT and 4.70% for the RPA. The reproducibility was evaluated with five independently prepared sample solutions, and the variation of the RRT and RPA of the characteristic peaks did not exceed 0.71% and 4.95%, respectively. The stability test was performed by analyzing of the same sample solution at different times (0, 2, 4, 6, 8, 12 and 24 h) and the RSDs of stability were below 0.91% for the RRT and 4.43% for the RPA. These results confirmed that the method of HPLC for the fingerprint analysis was valid and satisfactory (Table 1).

*Fingerprint analysis of FY capsule*

In order to establish the representative HPLC fingerprint of FY capsule, 10 different batches of samples were analyzed, and each chromatogram was used to construct the reference chromatograms. On the premise of achieving optimal chromatographic resolution and peak patterns, 28 peaks were screened out as characteristic peaks (Figure 2A). Similarity is an important parameter for HPLC fingerprinting and comes from the correlation coefficient of the original data. Correlation coefficients of 10 different batches of FY capsule were calculated by the Similarity Evaluation System for Chromatographic Fingerprints of TCM (Version 2004) (Figure 2B). The similarities of the chromatograms of the 10 samples were evaluated by comparing each sample with the reference fingerprint. As shown in Table 2, the quality of sample 1 was lower than that of the other samples. Despite this, the lowest similarity value was only 0.985, which indicated that these samples had a high similarity and there were no obvious differences in the quality of the 10 batches of FY capsule. Despite this, the lowest similarity value was only 0.985, which indicated that these samples had a high similarity and there were no obvious differences in the quality of the 10 batches of FY capsule.

*Identification of constituents in FY capsule*

According to MS/MS data obtained by collision-induced dissociation, fourteen components were unambiguously identified by the comparison of their retention times, MS data and UV spectra with the reference constituents. Table 3 displayed the data of MS/MS of the main components. All the components were detected in negative mode. To further illustrate the characteristic peaks and chemical constitution of FY capsule, the characteristic chromatograms were compared with the chromatograms of reference compounds (Figure 3). According to the fragmentation behavior of the reference standards and consistence in retention times, there were fourteen peaks among the 28 characteristic peaks that were unambiguously identified: gallic acid, quercitrin, quercetin, isoquercitrin, syringin, caffeic acid, myricetin, rosmarinic acid, lithospermic acid, syringic acid, methyl gallate, ellagic acid, protocatechuic acid, and chlorogenic acid (Table 3 and Figure 3).

*Calibration curves and the limit of detection*

The mixed standard stock solution containing fourteen components was diluted to appropriate concentrations for plotting the calibration curves. All calibration curves were plotted based on linear regression analysis of the integrated peak areas (y) versus concentrations (x, μg·mL^−1^) of the fourteen marker constituents in the standard solution at seven different concentrations. The regression equations, correlation coefficients, and linear ranges for the analysis of the fourteen marker constituents are shown in Table 4. All the analyses showed good linearity (R^2 ^> 0.9991) in a relatively wide concentration range. The limit of detection value (LOD) was calculated as the amount of the injected sample which gave a signal-to-noise ratio of 3 (S/N = 3). The LOD values of the method for the fourteen components are also listed in Table 4.

*Precision, accuracy and stability*

The relative standard deviation (RSD) was taken as a measure of precision and accuracy. The precision of the method was evaluated through performing intra-day and inter-day assays at three concentrations during a single day and on five consecutive days, respectively. As shown in Table 5, the overall intra- and inter-day variations were between 0.01-0.86% and 0.02-0.74%.

The accuracy tests were carried out using a recovery test by adding three different quantities (low, medium and high) of the fourteen standards into samples. The resultant samples were processed as described in Section 2.4 and analyzed using the developed HPLC method. The quantity of each analyte was subsequently obtained from the corresponding calibration curve. The percentage recoveries were calculated according to the following equation: (detection amount - original amount)/added amount × 100%. As a result of calculation, the recovery of all fourteen tested bioactive constituents was within the range of 97.81-102.54% with RSD less than 2.14%. The results of precision and recovery test indicate that the method has good precision and accuracy.

*Quantification of fourteen analytes in commercial FY capsule samples*

The proposed HPLC-DAD method was successfully applied to the simultaneous determination of the fourteen marker constituents in ten commercial production batches of FY capsule. All the contents were summarized in Table 6. As shown in Figure 2A and Table 5, under the analytical conditions, the fourteen marker constituents (GA, QI, QE, IQ, PA, CA, MY, RA, LA, SA, MG, EA, SY, and CHA) in FY can be sufficiently resolved and separated, which is suitable for the routine analysis and can contribute to quality control of commercial FY capsule.

## Conclusion

In the present research, chromatographic fingerprint analysis and simultaneous quantitative determination of fourteen marker components in FY capsule were established by HPLC-DAD. Twenty-eight characteristic fingerprint peaks were selected to evaluate the similarities of the 10 batches of FY capsule. HPLC-DAD-ESI-MS/MS was carried out to determination the structures of characteristic common peaks. Compared with their mass spectra and retention behaviour with reference standards or literature data, the structures of fourteen characteristic constituents were identified and quantitatively determined. 

The results clearly demonstrated that the proposed method was reasonable in linearity, repeatability, precision, stability and recovery, therefore, was fit for the routine analysis of FY capsule. The HPLC fingerprint analysis and the precise quantity of the marker components in the formula could provide valuable quantitative information for the quality assessment of FY capsule. Furthermore, the revelation of major constituents lays the groundwork for the deep study on further screening of the active ingredients in FY capsule.
